# Evaluating the Attraction of Scenic Spots Based on Tourism Trajectory Entropy

**DOI:** 10.3390/e26070607

**Published:** 2024-07-18

**Authors:** Qiuhua Huang, Linyuan Xia, Qianxia Li, Yixiong Xia

**Affiliations:** 1 Department of Geography Information Science, School of Geography and Tourism, Huizhou University, Huizhou 516007, China; huangqiuhua2000@hzu.edu.cn; 2 Department of Remote Sensing and GIS, School of Geography and Planning, Sun Yat-sen University, Guangzhou 510006, China; 3 Foshan Surveying Mapping And Geoinformation Research Institute, Foshan 528000, China; xiayx1@cug.edu.cn

**Keywords:** tourism trajectory, scenic spot, information entropy, attraction evaluation, experience index

## Abstract

With the development of positioning technology and the widespread application of mobile positioning terminal devices, the acquisition of trajectory data has become increasingly convenient. Furthermore, mining information related to scenic spots and tourists from trajectory data has also become increasingly convenient. This study used the normalization results of information entropy to evaluate the attraction of scenic spots and the experience index of tourists. Tourists and scenic spots were chosen as the probability variables to calculate information entropy, and the probability values of each variable were calculated according to certain methods. There is a certain competitive relationship between scenic spots of the same type. When the distance between various scenic spots is relatively close (less than 8 km), a strong cooperative relationship can be established. Scenic spots with various levels of attraction can generally be classified as follows: cultural heritage, natural landscape, and leisure and entertainment. Scenic spots with higher attraction are usually those with a higher A-level and convenient transportation. A considerable number of tourists do not choose to visit crowded scenic destinations but choose some spots that they are more interested in according to personal preferences and based on access to free travel.

## 1. Introduction

The attraction of a scenic spot can be defined as the degree to which it meets tourist expectations in terms of entertainment opportunities, food and accommodation, cultural richness, natural beauty, and other convenient facilities [1]. The concept of “tourism attraction” proposed by Formica, S. is a good tool for exploring supply–demand relationships [2]. The attraction of a destination is defined as “an individual’s perceived ability be provided by a destination that meets their special vacation needs” [3].

Although destination attraction has become one of the most popular topics in tourism research [4], the composition of destination attraction is complex, and its basic logic has not been fully explained [5]. The attraction of tourist destinations is the driving force for tourists to perceive value [6]. The most important matter is that the attraction of a destination encourages the public to visit and spend time there, which is foundational for encouraging tourist loyalty. Destination attraction also plays a crucial role in determining destination competitiveness [7].

Several researchers have attempted to determine the attraction of a destination and the factors that influence tourist decision-making processes in order to assess whether a region is an ideal vacation destination. The attractiveness of a region not only depends on its corresponding place and the characteristics of the local population but also on the cognitive image of the destination perceived by tourists [8]. Most researchers evaluate the attraction of tourism based on some factors of the destination, that is to say, based on previous evaluations, and rarely evaluate the attraction of tourism based on the behavior of tourists. This study evaluated the attraction of scenic spots based on the behaviors of tourists derived from tourism trajectory data; that is, it provides a post-evaluation.

### 1.1. Post-Evaluation of Attraction

This study uses trajectory data relating to tourists to evaluate the attraction index and tourist experience index of the Sanya Scenic Area. This index was constructed after examining tourism behaviors and was, therefore, a post-evaluation measure. This study mainly used information entropy and linear normalization methods to calculate the attraction of scenic spots and the tourist experience index and then classified the results. Based on the classification, some suggestions were proposed. Finally, the network relationships between scenic spots were displayed based on the spatial location information concerning tourism trajectories.

A post-evaluation can provide a reference for other tourists to understand the qualities and attractiveness of scenic spots. In this way, other tourists can better choose the scenic spots that they are interested in and avoid unsatisfactory travel experiences. Through post-evaluation, the managers of scenic spots can understand the true feelings and opinions of tourists. This helps managers and staff understand their shortcomings and improve service quality. They can make improvements based on feedback from tourists, and enhance the attractiveness and reputation of a scenic spot.

### 1.2. Outline of the Article

This article is organized into the following five sections:

Section 1 serves as the introduction. The post-evaluation of attraction is presented, and the article is outlined.

Section 2 presents the literature review. It presents the concepts of destination attraction, the application of GPS in tourism, the use of network analysis in tourism, and the application of information entropy.

Section 3 outlines the methodology. It presents the study area and data obtained, as well as the conceptual framework, information entropy, and linear normalization.

Section 4 presents the results. It presents the identification of local tourists and visiting tourists, the attraction index of scenic spots, the tourist experience index, and the network relationship between scenic spots.

Section 5 provides the conclusions and discussion. It presents a comprehensive overview of the study’s results and their significance and provides insights into the potential implications and applications of the work.

## 2. Literature Review

### 2.1. The Destination Attraction

The attraction of tourism has been modeled in different contexts, but few have attempted to examine the overall attraction of a city from the perspective of tourists. The factors that attracted tourists the most were investigated with the aim of illustrating the attraction of urban tourism [9]. The relationships between destination attraction, satisfaction, the sense of reliving, and loyalty were explored among American tourists who experienced Silk Road tourism in Uzbekistan [10]. The determinants of the attraction of hot spring tourism destinations were explored in Taiwan from a demand-side perspective [11]. Themes, products, and designs were identified as the three most important attributes that contribute to enhancing the attraction of salt industry destinations and influencing the decision-making processes of tourists. The combination of theme and design was considered the most prominent feature of the attraction of salt heritage sites [12]. The effects of cognitive and affective images were examined in relation to destination attraction [13]. A study attempted to identify the determining factors of tourist destination attraction based on tourist expectations, experiences, and satisfaction with tourism-related attributes [14].

Some researchers have studied the attraction of industrial tourism, especially in relation to factory tourism [15]. Finally, exploratory factor analysis was conducted to determine the most important factors involved in the attractiveness of tourist destinations, which were outlined as follows: tourism infrastructure, historical and cultural attractors, natural attractors, and communication facilities and lifestyle [16]. The uniqueness of forest landscapes and unique climate phenomena are the two most important factors determining the attractiveness of forest leisure tourism [17]. Paying attention to their independence is the main indicator determining tourism attractiveness [18].

A unique model designed to capture the antecedents of local attractiveness was developed in the context of crowded tourism hotspots [19]. The relative attractiveness of competing tourist destinations and the views of individual tourists have been evaluated in relation to vacation destinations [20]. Every improvement and development of attraction variables, such as the location and facilities provided by scenic spots, will have an increasing impact on the intentions of tourists to revisit a destination [21]. The winners in the tourism industry will be those countries with attractive tourist destinations that attract large numbers of tourists [22].

### 2.2. The Application of GPS in Tourism

In recent years, the use of GPS trajectory data in tourist scenic spots has received significant attention. Researchers have explored various aspects of their application, including understanding tourist behavior, optimizing travel routes, and enhancing overall tourist experiences. Tourism mobility has attracted widespread attention from tourism scholars. In recent years, advances in information technology have enabled tourism researchers to obtain detailed information about the digital footprint of tourism. GPS devices are used to track different groups of tourists around scenic spots to determine where they have gone and how long they have visited specific locations. Tracking data have been combined with survey data to explore whether different types of tourists behave differently when visiting scenic spots. Most tourists follow similar routes and exhibit strong “main path inertia” [23,24].

Passive mobile GPS positioning records were used to study the travel behaviors of tourists, particularly the number of trips, time spent at each destination, and mode of transportation [25,26]. Bicycle trajectory data were used to analyze the spatio-temporal behavior of Chinese bicycle tourists in the Xizang Autonomous Region [27]. Global positioning system technology has been used to compare and contrast the behavioral patterns of first-time and repeat visitors to Hong Kong [28]. A data management platform was presented to handle heterogeneous data, including taxi data, social media data, and venue data, to analyze the behavior of tourists. This can provide insights into tourism trajectories, activities for governments, and businesses relevant to the tourism industry and will help predict future tourism trends [29].

GPS has also been used to study tourist destination selection [30,31,32], the similarity of tourist trajectories [33,34], the spatial behavior of tourists [35,36,37,38,39], and the transition model of tourism [40].

### 2.3. Network Analysis in Tourism

Network analysis has been used in many different disciplines of social sciences and fields, including organizational sociology, political science, and organization theory [41,42]. The use of network analysis in tourism may be more important than in other fields because a network refers to sets of elements and the ties and relations among them, which is relevant to the study of tourism.

In recent years, the tourism literature has witnessed an increasing amount of research focused on network analysis. Tourism research has studied supply, destinations, and policy systems through the use of network analysis, as well as analyzed the movements of tourists and their behavior patterns [43,44,45,46]. Network analysis between scenic spots can be further expanded to collaborative cooperation between scenic spots, collaborative marketing between scenic spots, and the sustainable development of the tourism industry [47,48].

### 2.4. The Application of Information Entropy

The concept of entropy originates from thermodynamics and is a function that describes the ability of an energetic system to work. It also has philosophical connotations and can be used to evaluate the status of other disciplinary systems. The connotation of entropy has been extended and applied in disciplines such as information theory and the life sciences. Shannon first introduced the concept of entropy to information theory in 1948 with the definition of information entropy. The information entropy proposed by Shannon has the advantage of being able to characterize the disorder of a system based on the uncertainty of the information source using probability measures and mathematical statistics. The emergence of information entropy marked the generalization of entropy. Subsequently, concepts such as social entropy, economic entropy, and ecological entropy also emerged.

In the case of researching cities, the use of information entropy is mainly reflected in the following applications: analyzing the spatio-temporal distribution of water resources in search of sustainable urban water resource management [49]; scenario analysis of environmental impacts in energy strategies to achieve reasonable urban energy planning [50]; landscape pattern change analysis to examine the relationships between landscape planning and chaotic entropy change [51]; and studying the evolution of urban household energy consumption structures [52]. Land use information entropy was used to analyze the dynamic changes and degree of transformation of various land use types within a certain period of time [53]. However, few studies have applied information entropy to research to tourism behavior. Based on previous research, this article applied information entropy to study the attraction of scenic spots, and the results of the final analysis can be used to assist tourists in making decisions.

## 3. Methodology

In this study, information entropy and linear normalization were used to evaluate the attraction of scenic spots, and the experience index of tourists was obtained through the use of GPS trajectories.

### 3.1. Study Area and Data

**Figure 1:**
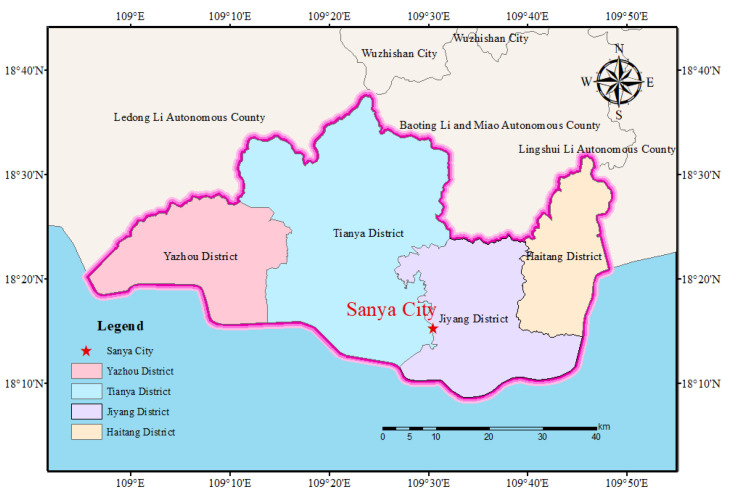
Location of the study area.

Sanya City is the second largest city in Hainan Province. It is located at the southernmost point of Hainan Island, covering a geographic extent from 18°09′34″ to 18°37′27″ N and from 108°56′30″ to 109°48′28″ E (as shown in Figure 1). The city is 91.6 km long from east to west and 51.8 km wide from north to south. Sanya City has a total land area of 1921 square kilometers and a total sea area of 3226 km^2^.

**Figure 2:**
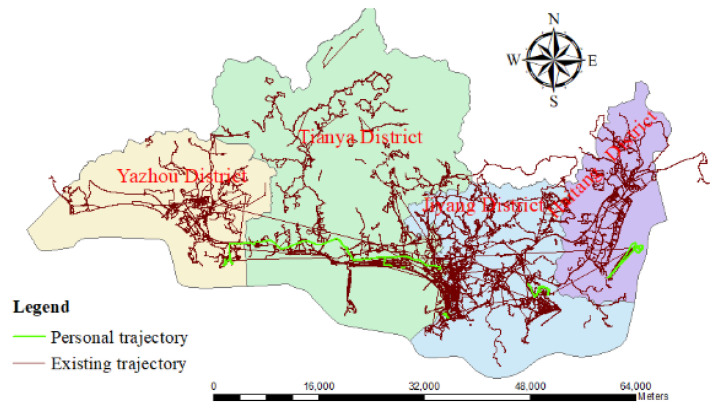
The spatial distribution of tourist trajectories.

The existing trajectory data for this study were provided by Six Legs (http://www.foooooot.com/accounts/login/, accessed on 10 July 2023), which is an outdoor travel software platform developed by Beijing Genghi Technology Co., Ltd. (Beijing, China). It provides users with powerful GPS trajectory recording capabilities, enables the convenient sharing of travel, allows diverse offline map downloads, and can be used to browse millions of outdoor travel routes at any time. The personal trajectory section was collected by the author during her research trip to Sanya using the Six Legs app (as shown in Figure 2).

### 3.2. Conceptual Framework

**Figure 3:**
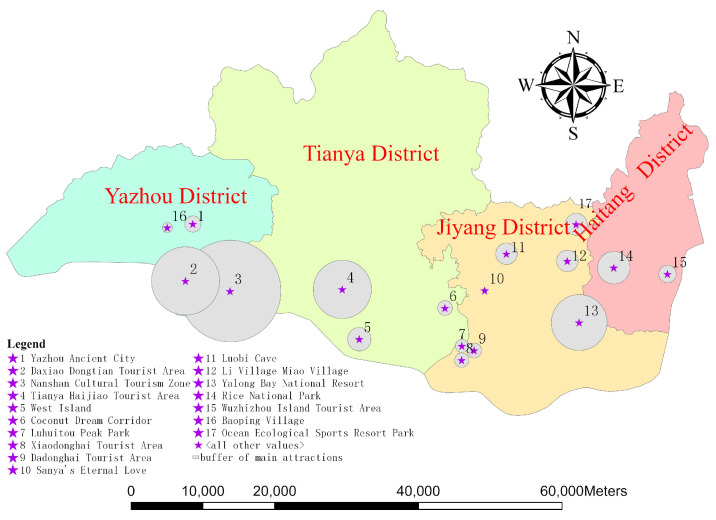
Buffer regions of each scenic spot.

Buffer radius of each scenic spot (*R_j_*): The buffer radius of each scenic spot was calculated based on its area *S_i_*, and then the buffer of each scenic spot was established to conform to its corresponding buffer radius. The specific buffer is shown in Figure 3. Because the area of each scenic spot is different, the buffer radii are also different.

(1) $$\;R_{j}=\sqrt{\frac{S_{j}}{\pi }},(j=1,2,\ldots \ldots ,17)$$

The scenic spots visited by trajectory (*Tr_i_S_j_*): Calculate whether each trajectory *Tr_i_* (*i* = 1, 2,……, 1553) intersects with the buffer of each scenic spot *S_j_* (*j* = 1, 2,……, 17). If it intersects, it is considered that trajectory *Tr_i_* has visited the scenic spot *S_j_* and *Tr_i_S_j_* = 1; otherwise, *Tr_i_S_j_* = 0. A trajectory can visit 0 or more scenic spots. Calculate the number of scenic spots visited by each trajectory and the specific scenic spots visited.

The number of visiting trajectories for each scenic spot (*N_Tr_S_j_*): Generate buffers (as shown in Figure 3) using the area of each scenic spot, and then perform spatial overlay analysis on all trajectories and the buffers. The number of trajectories *N_Tr_S_j_* that intersect with the buffer of each scenic spot is the number of visiting trajectories for that scenic spot *S_j_*.

(2) $$\;N_{Tr}S_{j}=\sum_{i=1}^{1553}(Tr_{i}S_{j}),(j=1,2,\ldots \ldots ,17)$$

The number of scenic spots for each trajectory (*NSTr_i_*): Perform spatial overlay analysis on all trajectories and the buffers. *NSTr_i_* is the number of scenic spots visited by trajectory *Tr_i_*, and the calculation formula is shown below, and the final result of this value ranges from 0 to 7; for the convenience of its subsequent representation, this value is represented by *Sm* (*m* = 0, 1, 2, …, 7).

(3) $$\;NSTr_{i}=\sum_{j=1}^{17}(Tr_{i}S_{j}),(i=1,2,\ldots \ldots ,1553)$$

Number of trajectories corresponding to the number of scenic spots visited by a trajectory (*N_Tr_Sm*): After calculating the number of scenic spots *Sm* visited by each trajectory, the number of trajectories *N_Tr_Sm* corresponding to the number of scenic spots *Sm* visited by a trajectory is calculated. Let *N_Tr_Sm* be the variable with an initial value of 0; the calculation formula is shown below, and the specific distribution is shown in Table 1.

(4) $$\;N_{Tr}Sm(m=0,1,2,\ldots ,7)=\left\{ \begin{array}{l} if\\ N_{Tri}S=m,(i=1,2,\ldots \ldots ,1553),N_{Tr}Sm+=1\\ else\\ N_{Tr}Sm=N_{Tr}Sm \end{array}\right.$$

In this study, of a total of 1553 trajectories, nearly half (a total of 775) did not visit any of the scenic spots listed. These trajectories are considered non-sightseeing visits. The remaining 778 trajectories visited at least one scenic spot. From the distribution table shown above, it can be seen that most trajectories (totaling 620) only visited one scenic spot. As the number of scenic spots increases, the corresponding number of trajectories gradually decreases. Only one trajectory visited 6 scenic spots, and one trajectory visited 7 scenic spots; however, it is worth noting that the two trajectories may represent continuous records across multiple days.

The total visiting number of all scenic spots (*ToS*): Multiply the number of scenic spots (*Sm*) by the corresponding number of trajectories (*N_Tr_Sm*), add them up, and finally calculate the total visiting number of all scenic spots (*ToS*). The calculation formula is shown below, and the result is 1025.

(5) $$ToS=\sum_{m=0}^{7}Sm*N_{Tr}Sm,(m=0,1,2,\ldots ,7)$$

The average visiting number of each scenic spot (*N_A_*): By dividing the total number of visits to all scenic spots (*ToS*) by the number of scenic spots 17, the average visiting number of each scenic spot (*N_A_*) can be calculated using the formula presented below, and the result is approximately 60.

(6) $$N_{A}=ToS/17$$

The average visiting rate of each scenic spot (*R_A_*): The average visiting rate of each scenic spot is calculated by dividing the average visiting number of each scenic spot (*N_A_*) by the total number of trajectories (1553). The calculation formula is shown below; the result is approximately 3.86%. If the visiting rate of a scenic spot is higher than 3.86%, it is considered to have a relatively high visitation rate.

(7) $$R_{A}=(N_{A}/1553)*100$$

In this study, there were a total of 7 scenic spots with a relatively high visiting rate. The visiting rates of 7 scenic spots were ranked from high to low: the Coconut Dream Corridor (13%), the Yalong Bay Tropical Paradise Forest Park (9%), the Tianya Haijiao Tourist Area (6%), the Dadonghai Tourist Area (6%), the Nanshan Cultural Tourism Zone (6%), the Luhuitou Peak Park (5%), and the Daxiao Dongtian Tourist Area (4%).

Number of tourists visiting each scenic spot (*N_To_S_j_*): Calculate the number of trajectories and the corresponding trajectories for visitations to each scenic spot and analyze the tourist code of the trajectories (*ToCTr_i_*). If the trajectory belongs to various tourists, accumulate the quantity; otherwise, do not count it. Use the summary function of Excel to count the number of different tourists visiting the same scenic spot.

### 3.3. Information Entropy

For an uncertain system, if the state characteristics are represented by a random variable *X*, for a discrete random variable, set the value of *X* to $X=\left\{x_{1},x_{2},\ldots x_{n}\right\}(n\geq 2)$, and the probability corresponding to each value is $P=\left\{p_{1},p_{2},\cdots p_{n}\right\}(0\leq p_{i}\leq 1,i=1,2,\cdots ,n)$, $\sum p_{i}=1$, then the information entropy of the system is shown below:

(8) $$\;\;S(X)=-\sum_{i=1}^{n}p_{i}\log_{2}(p_{i})$$

Generally speaking, there is just one category that dominates the system. Assuming that this state is an extreme state, the minimum value of information entropy occurs as follows: $S_{\min}=0$; on the contrary, if the number of various categories in the system is equal, then $p_{1}=p_{2}=\cdots =p_{n}=1/n$, $S_{\max}=\log_{2}n$. However, in practice, both of these situations are basically non-existent. Generally, the size of information entropy is situated between them, and its size directly reflects the complexity of the system.

### 3.4. Linear Normalization

Min–max normalization, for example, can be used to transform a value x of a numeric attribute A to $x^{\prime }$ in the range [0,1] by computing

(9) $$x^{\prime }=\frac{x-\min_{A}}{\max_{A}-\min_{A}},$$

where $\min_{A}$ and $\max_{A}$ are the minimum and maximum values of attribute A.

## 4. Results

### 4.1. The Identification of Local Tourists (Lo-Tour) and Visiting Tourists (NLo-Tour)

Whether a tourist is a local tourist or a visiting tourist can be determined based on the following factors: the degree of concentration (most trajectories overlap) and dispersion (trajectories are scattered without overlapping) of their trajectories, the number of scenic spots visited, and the distribution of trajectory times. In this study, individuals whose trajectory time span is less than or equal to 15 days are considered visiting tourists, while those whose time span exceeds 15 days are considered local tourists. If the number of visiting scenic spots is 0, it is considered a trip to a non-scenic spot; if it is greater than 0 but less than 5, it is considered to be a trip with a few scenic spots; if it is greater than or equal to 5, it is considered to be a trip with multiple scenic spots. In this study, the average number of trajectories per tourist is 2.6. In this study, only tourists with three or more personal trajectories were analyzed, mainly focusing on the following aspects: analysis of tourist attractions, trajectory distribution, and trajectory generation time.

Finally, local tourists and visiting tourists are divided into the following categories: non-scenic spot concentration, few scenic spots concentration, and multiple scenic spots concentration, as well as non-scenic spot dispersion, few scenic spots dispersion, and multiple scenic spots dispersion. In this study, 125 tourists were satisfied in that the number of personal trajectories was greater than or equal to 3. The final classification results are shown in Table 2 below.

The following conclusions can be drawn from Table 2:

(1) Among the 125 tourists, 67 were local tourists, and 58 were visiting tourists, with local tourists slightly exceeding visiting tourists.
(2) For tourists that visit zero scenic spots, the trajectory distribution of local and visiting tourists is often more concentrated, which means that most local tourists choose to relax during their rest time and travel around their homes. However, visiting tourists choose to relax and explore the surrounding areas near the hotel.
(3) For tourists that visit a few scenic spots, the trajectories of local tourists are often scattered, while the trajectories of visiting tourists are often concentrated. Considering the familiarity that local tourists have with the area, the visiting range will be larger; on the other hand, visiting tourists will choose hotels with many scenic spots to stay in, and then choose places near these hotels for leisure tourism, leading to a relatively concentrated trajectory distribution.
(4) For tourists that visit many scenic spots, whether local or visiting, there is a relatively scattered trajectory distribution, which is directly related to the number of scenic spots and conforms to a major characteristic of multiple scenic spots.
(5) The concentration and dispersion distribution differences between local tourist trajectories are relatively small. However, for visiting tourists, what is more obvious is that the trajectory of most tourists is concentrated.
(6) In terms of the number of scenic spots visited, local and visiting tourists have the highest proportion of visiting only a few scenic spots, about 60%; followed by non-scenic spot, accounting for more than 20%; and multiple scenic spots, accounting for less than 20%. This indicates that over 20% of tourists choose non-scenic leisure tours.

In this study, the scenic spots visited by local tourists or visiting tourists were summarized; they are shown in Table 3. The following conclusions were drawn from the table:

(1) For local tourists, the Dadonghai Tourist Area, the Coconut Dream Corridor, the Luhuitou Peak Park, and the Yalong Bay Tropical Paradise Forest Park are the most popular scenic spots; Yazhou Ancient City is more likely to be repeatedly visited.
(2) For visiting tourists, the Nanshan Cultural Tourism Zone, the Tianya Haijiao Tourist Area, the Yalong Bay Tropical Paradise Forest Park, the Luhuitou Peak Park, and the Dadonghai Tourist Area are the most popular scenic spots; the Coconut Dream Corridor is more likely to be repeatedly visited.
(3) From the analysis results, it can be seen that local tourists have lower enthusiasm for the Nanshan Cultural Tourism Zone and Tianya Haijiao Tourism Area compared to visiting tourists.
(4) Yazhou Ancient City is a scenic spot that is frequently visited by local tourists but rarely visited by visiting tourists. It is speculated that this is because the scenic spot is not well known to the public.

**Figure 4:**
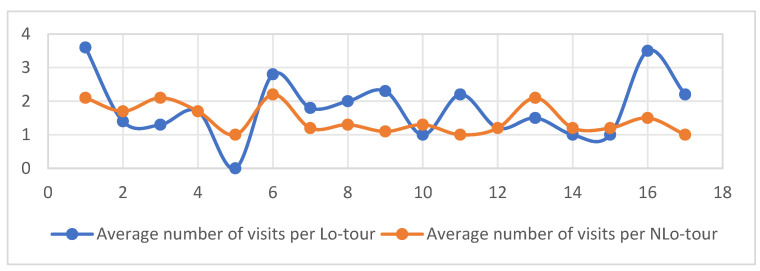
Average number of visits by Lo-tour and NLo-tour to the 17 scenic spots.

From Figure 4, it can be seen that the average number of visits by local tourists to various scenic spots is generally higher, while the average number of visits by visiting tourists is generally smaller, close to once. This distribution is in line with the characteristics of local and visiting tourists. Most visiting tourists only visit the scenic spots once and rarely repeat their visits to the same place.

### 4.2. Attraction Index of Scenic Spots

The results of the kernel density analysis can only indicate that a certain scenic spot has a relatively large number of trajectories, and it does not indicate that the scenic spot has high attraction and is definitely worth recommending to tourists. This is due to the fact that there is a situation where certain scenic areas are located near a tourist’s residence, and the trajectories of their frequent walks are recorded, resulting in a higher density value in the core of the scenic area.

#### 4.2.1. Regression Model

**Figure 5:**
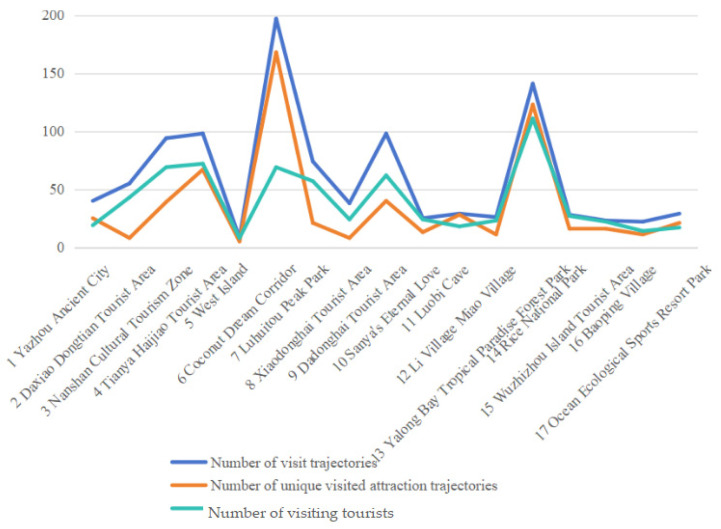
The number of trajectories and tourists relating to 17 scenic spots.

Generally speaking, the more visits and tourists a scenic spot attracts, the more popular it becomes. The Figure 5. shows the number of visitation trajectories, the number of trajectories visiting unique scenic spots, and the number of visiting tourists for the 17 scenic spots. There is a strong correlation between them, with peaks occurring in the same scenic spot.

In this study, an attraction regression model was created for each scenic spot using SPSS, as shown in Formula (10), where the dependent variable *Y* is the attraction of each scenic spot, and the other independent variables are the number of visiting trajectories (*X*_1_), the number of unique visited trajectories (*X*_2_), and the number of visiting tourists (*X*_3_) for each scenic spot.

(10) $$Y=0.153+0.066*X_{1}+0.001*X_{2}+0.006*X_{3}$$

#### 4.2.2. Tourism Entropy

In this study, the information entropy of each scenic spot was calculated using tourists visiting the same scenic spot as the characteristic variable. The probability of each tourist is equal to the number of times they visited the scenic spot divided by the total number of visits to the scenic spot. The entropy formula is used to calculate the entropy value of each scenic spot, and finally, linear normalization can be performed on the entropy value to distribute it between 0 and 1. The formula is shown below (11); *S(A)* is the tourism entropy of scenic spot *A*. For example, suppose there are five tourists visiting scenic spot *A*, with each tourist visiting the scenic spot two, one, three, one, and one times, respectively; the total number of visits to scenic spot *A* is eight, and the probabilities of five tourists appearing at the scenic spot $P=\left\{p_{1},p_{2},\cdots p_{5}\right\}(0\leq p_{i}\leq 1,i=1,2,\cdots ,5)$ are *P* = {2/8, 1/8, 3/8, 1/8, 1/8}, respectively, with a total probability of 1. By substituting the probability values of the five tourists into the information entropy formula, the information entropy value of scenic spot *A* can be calculated as follows:

(11) $$\;\;S(A)=-\sum_{i=1}^{n}p_{i}\log_{2}(p_{i})$$

#### 4.2.3. Comparative Analysis of Tourism Entropy and Regression Models

**Figure 6:**
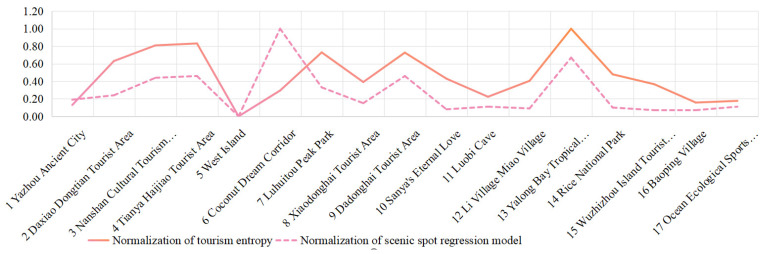
The scenic spot index of different scenic spots.

The results of the regression model and the tourism entropy were normalized linearly. The normalized results of each scenic spot are shown in Figure 6. From the graph, it can be seen that the distribution trends are basically the same, except for the abnormal location of the Coconut Dream Corridor. Here, there are too many repeated trajectories visiting the Coconut Dream Corridor by one tourist; therefore, the results calculated using the regression model exaggerate the attraction of this scenic spot, while the results produced via tourism entropy are more reasonable. The regression model may exaggerate the attraction index of a certain scenic spot when the same tourist undertakes repeated trajectories; however, this situation is not observed when using the tourism entropy value. From the comparison of the attraction index values of different scenic spots obtained via two calculation methods, it can be seen that the comparison of the attraction values of different scenic spots calculated using tourism entropy is more reasonable. Therefore, the normalized result of tourism entropy is used as an indicator of the attractiveness of scenic spots.

#### 4.2.4. Attraction Index Rating of Scenic Spots

Scenic spots are classified into four levels based on the attraction index; the intervals are as follows: level four (0–0.25), level three (0.25–0.5), level two (0.5–0.75), level one (0.75–1). The first and second levels each have three scenic spots, while the third and fourth levels have six and five scenic spots, respectively.

Each scenic spot is defined as natural, cultural heritage, or leisure and entertainment type based on its characteristics. The ticket prices and A-level ratings were collected for each scenic spot, as well as the final attraction rating of the scenic spots, as shown in Table 4. The A-level ratings of each scenic spot have a corresponding evaluation standard, and the tourism A-level was obtained from the official tourism website. From a typological perspective, each level basically covers three types, indicating a certain degree of competition among scenic spots of the same level. The attraction of a scenic spot is not closely related to its price but has a strong correlation with its A-level, with higher levels of attraction generally having higher A-levels.

**Figure 7:**
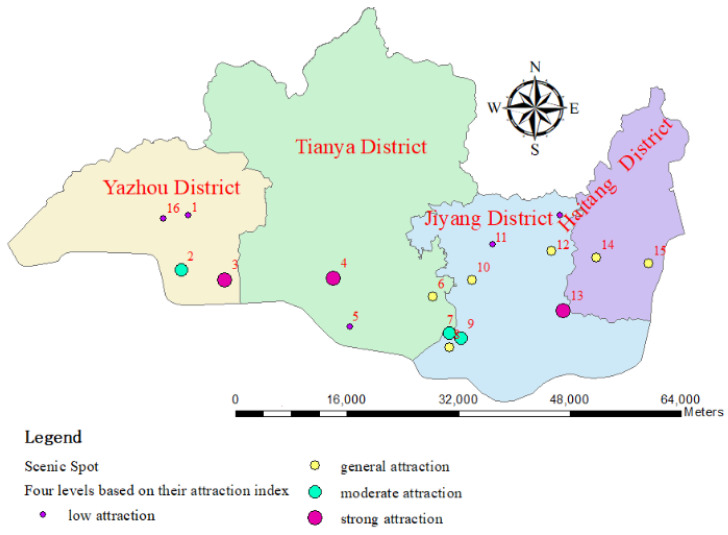
The visualization of scenic spots based on their attraction index.

Each scenic spot is visualized based on its attraction index, as shown in Figure 7. Based on the above analysis, the following conclusions can be drawn:

(1) Scenic spots with higher A-level generally have higher attraction, meaning that the attraction of scenic spots is significantly influenced by their level.
(2) A-level or above scenic spots will not decrease their attraction due to high ticket prices, which means that the attraction of scenic spots will not be significantly affected by ticket prices.
(3) Due to inconvenient transportation, the attraction of some A-level and above scenic spots has been seriously affected, which means that the attraction of scenic spots is more significantly affected by transportation convenience.

### 4.3. Tourist Experience Index

We cannot assume that tourists have had a tourist experience just because they have a greater number of trajectories, as some tourists may often take walks at nearby scenic spots and record and upload these trajectories. Although some tourists have a large number of tourism trajectories, most of which are repetitive, they have not visited multiple regions and do not have much tourism knowledge about the city.

In this study, the normalized results relating to tourist information entropy were used as the experience index to measure tourist experience values. The scenic spots visited by the same tourist were taken as the characteristic variable to calculate the information entropy of each tourist. The probability of each scenic spot visited by a certain tourist is equal to the number of times the scenic spot was visited divided by the total number of times all scenic spots visited by the same tourist. The formula is shown below (12); *S(T)* is the tourist experience index of tourist *T*. For example, suppose there are five scenic spots visited by tourist *T*, with each scenic spot visited two, one, three, one, and one times, respectively; the total number of times all scenic spots were visited by tourist *T* is eight, and the probabilities of five scenic spots visited by tourist *T* $P=\left\{p_{1},p_{2},\cdots p_{5}\right\}(0\leq p_{i}\leq 1,i=1,2,\cdots ,5)$ are *P* = {2/8, 1/8, 3/8, 1/8, 1/8}, respectively, with a total probability of 1.

(12) $$\;\;S(T)=-\sum_{i=1}^{n}p_{i}\log_{2}(p_{i})$$

In this study, the tourist experience index was used to measure the amount of information that tourists have about scenic spots in Sanya. Therefore, the experience index of tourists who have not visited a scenic spot is considered to be 0, indicating that they are not familiar with the city’s scenic spots. The average number of trajectories per tourist was 2.6, as calculated previously; thus, only the tourists with a number of trajectories greater than 3 were used to calculate information entropy. In this sample, a total of 125 tourists met these conditions.

**Figure 8:**
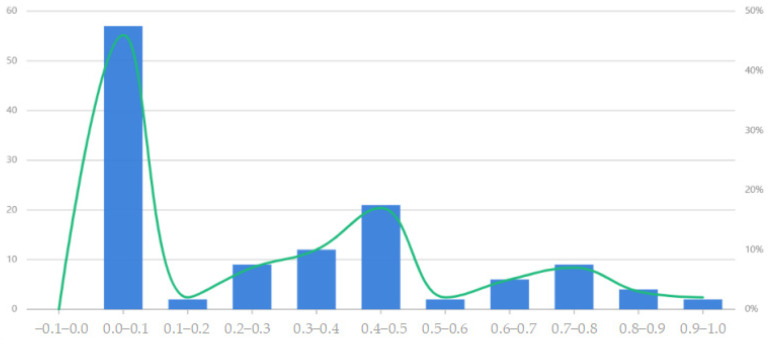
Distribution of tourist experience index.

The tourist experience index is divided into four levels at equal intervals, and the specific rating range and corresponding number of tourists are shown in Table 5:

From Table 5, it can be seen that approximately 80% of the tourist experience index is distributed in the range of [0.0–0.5]. From Figure 8, it can be seen that there is still a lot of development space for these scenic spots, and nearly 80% of tourists can become potential visitors to the scenic spots. That is to say, there is still a lot of room for improvement in terms of the services offered by scenic spots. Scenic spots can be upgraded and transformed to enhance their attractiveness to tourists, allowing these scenic spots to fully leverage their cultural, natural, economic, social, and educational value.

### 4.4. Network Relationship between Scenic Spots

**Figure 9:**
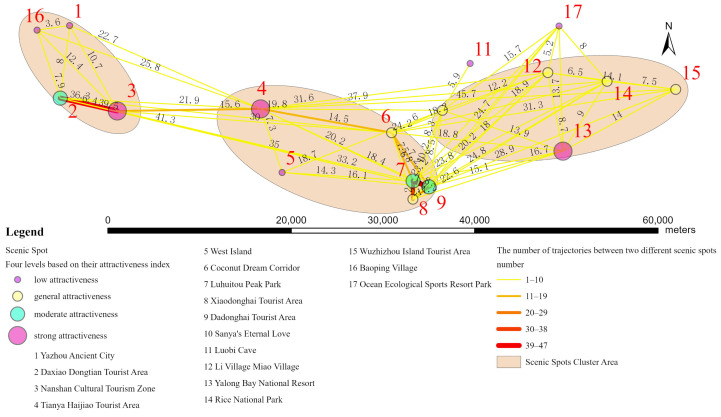
Network relationship between scenic spots.

The network relationship between scenic spots was created by tracking tourism trajectories, as shown in Figure 9. In Figure 9, points of different colors and sizes represent scenic spots with different levels of attractiveness. The data on the line represent the distance between the two connected scenic spots, and the color and thickness of the line represent the number of trajectories connecting the two scenic spots. It can be seen that the closer the distance between scenic spots, the more trajectories connect them. According to the spatial distribution of scenic spots and the network relationship between them, scenic spots can be divided into three cluster areas, each consisting of scenic spots with higher levels of attraction and a certain number of neighboring scenic spots. From left to right, based on the main characteristics of the scenic spots, the cluster area can be divided into cultural heritage areas, natural landscape areas, and leisure and entertainment areas.

**Figure 10:**
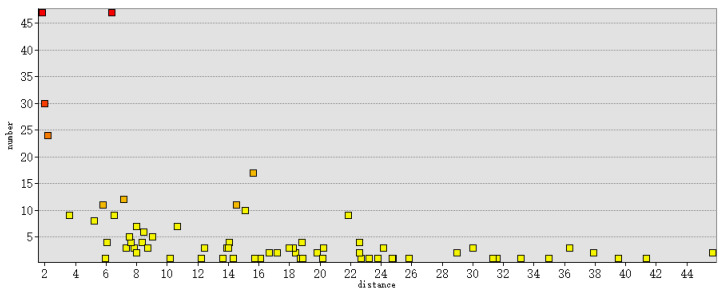
The number of connection trajectories corresponding to different distances (unit: km).

Figure 10 shows the number of connection trajectories corresponding to different distances between scenic spots. From the distribution shown in the graph, the distance between scenic spots can be divided into four levels: (0–8), (8–16), (16–24), and (24–+∞), in kilometers. From the graph, it can be seen that when the distance between scenic spots is less than 8 km, there are more connecting trajectories between them, with some scenic spots having more than 45 connecting trajectories. When the connection distance is between 8 and 16 km, the number of connection trajectories approaches 20. When the distance is between 16 and 24 km, the number of connection trajectories approaches 10. When the distance is greater than 24 km, the number of connection trajectories is relatively small, usually less than five.

## 5. Conclusions and Discussion

The scenic spots visited by each trajectory were identified by utilizing tourism trajectory data and the spatial buffer zone of each scenic spot. At the same time, detailed information, such as the number of trajectories and tourists visiting each scenic spot, was also obtained. From the attribute information of each trajectory, the author status of the trajectory can be determined, and then the trajectory distributions of different tourists and the scenic spots visited can be summarized.

Both the attractiveness of scenic spots and the travel experiences of tourists cannot be simply judged by analyzing the number of trajectories or scenic spots visited. This study used the normalized results of information entropy to evaluate the attractiveness of scenic spots and the experience index of tourists. Tourists and scenic spots were selected as the probability variables to calculate information entropy, and the probability values of each variable were calculated according to certain methods. In this study, various terms involved in the calculation process were defined, and their calculation formulas were provided.

The scenic spots and their various levels of attractiveness generally covered three types: cultural heritage, natural landscape, and leisure and entertainment. That is to say, the same types of scenic spots have a clear distribution in terms of levels of attraction, while different types of scenic spots have a certain degree of parallelism in their attraction distribution. When tourists travel and choose scenic spots, there is a certain degree of exclusivity among the same type of scenic spots. Nearly 80% of tourists have experience values below 0.5, indicating that there is still a lot of room for improvement in terms of their experience values. We can attract more tourists to visit scenic spots by increasing advertising and service improvement, thereby enhancing their experience value.

By examining the network relationship between scenic spots, we can see that when the distance between different scenic spots is relatively close (less than 8 km), a strong cooperative relationship can be established. As the distance increases, the cooperative relationship gradually weakens. Scenic spots with higher attractiveness often have higher A-level ratings or more convenient transportation, and the correlation between the attractiveness and whether it is free or ticketed is not significant. The development of nearby scenic spots can be furthered to leverage the clustering effect of tourism resources, and the tourism market can be further developed through complementary types of tourism resources.

Future works focusing on the network relationship between scenic spots have enormous potential in terms of enhancing connectivity, collaboration, and marketing. By utilizing these opportunities, scenic spots can create a more interconnected and seamless travel experience for tourists, improve operational efficiency for managers, and contribute to the development of the tourism industry. Moreover, the network analysis results between existing scenic spots can be used to develop new tourism resources and arrange tourism service facilities.

## Figures and Tables

**Table 1:** Number of trajectories corresponding to the number of scenic spots.

Number of scenic spots (*Sm*)	0	1	2	3	4	5	6	7
Number of trajectories (*N_Tr_Sm*)	775	620	95	44	15	2	1	1

**Table 2:** The distribution of local or visiting tourists.

		Concentration	Dispersion	Total (Proportion%)
Local tourists (67 people)	Non-scenic spot	12	4	16 (24%)
	Few scenic spots	18	21	39 (58%)
	Multi scenic spots	3	9	12 (18%)
	Total	33	34	67
Visiting tourists (58 people)	Non-scenic spot	11	1	12 (21%)
	Few scenic spots	21	16	37 (64%)
	Multi scenic spots	1	8	9 (15%)
	Total	33	25	58

**Table 3:** The distribution of local and visiting tourists for different scenic spots.

	Number of Visits by Local Tourists	Number of Local Tourists	Average Number of Visits per Local Tourist	Number of Visits by Visiting Tourists	Number of Visiting Tourists	Average Number of Visits per Visiting Tourist
1 Yazhou Ancient City	18	5	3.6	15	7	2.1
2 Daxiao Dongtian Tourist Area	17	12	1.4	10	6	1.7
3 Nanshan Cultural Tourism Zone	19	15	1.3	33	16	2.1
4 Tianya Haijiao Tourist Area	26	15	1.7	27	16	1.7
5 West Island	0	0	0.0	3	3	1.0
6 Coconut Dream Corridor	34	12	2.8	24	11	2.2
7 Luhuitou Peak Park	31	17	1.8	17	14	1.2
8 Xiaodonghai Tourist Area	22	11	2.0	4	3	1.3
9 Dadonghai Tourist Area	48	21	2.3	16	14	1.1
10 Sanya’s Eternal Love	5	5	1.0	5	4	1.3
11 Luobi Cave	20	9	2.2	1	1	1.0
12 Li Village Miao Village	7	6	1.2	6	5	1.2
13 Yalong Bay Tropical Paradise Forest Park	24	16	1.5	25	12	2.1
14 Rice National Park	2	2	1.0	6	5	1.2
15 Wuzhizhou Island Tourist Area	3	3	1.0	6	5	1.2
16 Baoping Village	7	2	3.5	9	6	1.5
17 Ocean Ecological Sports Resort Park	20	9	2.2	1	1	1.0

**Table 4:** Attraction index, ticket price, A-level, and types of different scenic spots.

Scenic Spot	Attraction Index	Ticket Price Unit: CNY (A-Level)	Types of Scenic Spots (1: Natural Landscape. 2: Cultural Heritage. 3: Leisure and Entertainment)
5 West Island	0.00	95 (4A)	3
1 Yazhou Ancient City	0.13	0	2
16 Baoping Village	0.16	0	2
17 Ocean Ecological Sports Resort Park	0.18	0	1
11 Luobi Cave	0.22	0	1
6 Coconut Dream Corridor	0.29	0	3
15 Wuzhizhou Island Tourist Area	0.37	140 (5A)	3
8 Xiaodonghai Tourist Area	0.39	0	3
12 Li Village Miao Village	0.40	0	2
10 Sanya’s Eternal Love	0.43	280	2
14 Rice National Park	0.48	30 (4A)	1
2 Daxiao Dongtian Tourist Area	0.63	0 (5A)	1
9 Dadonghai Tourist Area	0.73	0 (4A)	3
7 Luhuitou Peak Park	0.73	0 (4A)	1
3 Nanshan Cultural Tourism Zone	0.81	122 (5A)	2
4 Tianya Haijiao Tourist Area	0.83	0 (4A)	3
13 Yalong Bay Tropical Paradise Forest Park	1.00	148 (4A)	1

**Table 5:** Tourist experience index grading.

Experience Index Interval	Number of Tourists	Proportion (%)
0–0.25	62	0.50
0.26–0.50	39	0.31
0.51–0.75	13	0.10
0.76–1	11	0.09
Total (unit: person)	125	1.00

## Data Availability

The data used in this study are available upon request. However, due to privacy and confidentiality concerns, certain restrictions may apply to the availability of the data. Requests for access to the data can be made to the corresponding author.

## References

[1] Pearce D.G. (1979). Towards a geography of tourism. Ann. Tour. Res..

[2] Formica S. (2000). Destination Attractiveness as a Function of Supply and Demand Interaction. Ph.D. Dissertation.

[3] Hu Y., Ritchie J.R.B. (1993). Measuring Destination scenic spot: A Contextual Approach. J. Travel Res..

[4] Pike S. (2002). Destination image analysis: A review of 142 papers from 1973 to 2000. Tour. Manag..

[5] Edward M., George B. (2008). Tourism development in the state of Kerala, India: A study of destination scenic spot. Eur. J. Tour. Res..

[6] Ariya G., Wishitemi B., Sitati N. (2017). Tourism destination scenic spot as perceived by tourists visiting Lake Nakuru National Park, Kenya. Int. J. Res. Tour. Hosp..

[7] Buhalis D. (2000). Marketing the competitive destination of the future. Tour. Manag..

[8] Dimitrov P.M., Stankova M.Z., Vasenska I., Uzunova D. (2017). Increasing attractiveness and image recognition of Bulgaria as a tourism destination. Tour. Manag. Stud..

[9] Boivin M., Tanguay G.A. (2019). Analysis of the determinants of urban tourism scenic spot: The case of Québec City and Bordeaux. J. Destin. Mark. Manag..

[10] Raimkulov M., Juraturgunov H., Ahn Y. (2021). Destination scenic spot and memorable travel experiences in silk road tourism in Uzbekistan. Sustainability.

[11] Lee C.F., Ou W.M., Huang H.I. (2009). A study of destination scenic spot through domestic visitors’ perspectives: The case of Taiwan’s hot springs tourism sector. Asia Pac. J. Tour. Res..

[12] Wu T.C., Xie P.F., Tsai M.C. (2015). Perceptions of scenic spot for salt heritage tourism: A tourist perspective. Tour. Manag..

[13] Kim D., Perdue R.R. (2011). The influence of image on destination scenic spot. J. Travel Tour. Mark..

[14] Das D., Sharma S.K., Mohapatra P.K., Sarkar A. (2007). Factors influencing the attractiveness of a tourist destination: A case study. J. Serv. Res..

[15] Lee C.F. (2016). An investigation of factors determining industrial tourism scenic spot. Tour. Hosp. Res..

[16] Islam S., Hossain M.K., Noor M.E. (2017). Determining drivers of destination scenic spot: The Case of nature-based tourism of Bangladesh. Int. J. Mark. Stud..

[17] Lee C.F., Huang H.I., Yeh H.R. (2010). Developing an evaluation model for destination scenic spot: Sustainable forest recreation tourism in Taiwan. J. Sustain. Tour..

[18] Gearing C.E., Swart W.W., Var T. (1974). Establishing a measure of touristic scenic spot. J. Travel Res..

[19] Jacobsen J.K.S., Iversen N.M., Hem L.E. (2019). Hotspot crowding and over-tourism: Antecedents of destination scenic spot. Ann. Tour. Res..

[20] Cracolici M.F., Nijkamp P. (2009). The scenic spot and competitiveness of tourist destinations: A study of Southern Italian regions. Tour. Manag..

[21] Khairi M., Darmawan D. (2021). The Relationship Between Destination scenic spot, Location, Tourism Facilities, And Revisit Intentions. J. Mark. Bus. Res. (MARK).

[22] Blazeska D., Milenkovski A., Gramatnikovski S. (2015). The quality of the tourist destinations a key factor for increasing their scenic spot. UTMS J. Econ..

[23] East D., Osborne P., Kemp S., Woodfine T. (2017). Combining GPS & survey data improves understanding of visitor behaviour. Tour. Manag..

[24] Huang Q. (2019). A LBS Supported Mining System for Self-Help Tourism—A Chinese Case Study. DEStech Trans. Comput. Sci. Eng..

[25] Phithakkitnukoon S., Horanont T., Witayangkurn A., Siri R., Sekimoto Y., Shibasaki R. (2015). Understanding tourist behavior using large-scale mobile sensing approach: A case study of mobile phone users in Japan. Pervasive Mob. Comput..

[26] Huang Q., Xia L. (2016). Inspection of spatial-temporal behavior of backpackers in Beijing based on trajectory. Wirel. Pers. Commun. Int. J..

[27] Mou N., Liu Z., Zheng Y., Makkonen T., Yang T., Zhang L. (2022). Cycling in Tibet: An analysis of tourists’ spatiotemporal behavior and infrastructure. Tour. Manag..

[28] McKercher B., Shoval N., Ng E., Birenboim A. (2012). First and repeat visitor behaviour: GPS tracking and GIS analysis in Hong Kong. Tour. Geogr. Int. J. Tour. Space Place Environ..

[29] Krataithong P., Anutariya C., Buranarach M. (2022). A Taxi Trajectory and Social Media Data Management Platform for Tourist Behavior Analysis. Sustainability.

[30] Li Y., Yang L., Shen H., Wu Z. (2019). Modeling intra-destination travel behavior of tourists through spatio-temporal analysis. J. Destin. Mark. Manag..

[31] Grinberger A.Y., Shoval N., McKercher B. (2014). Typologies of tourists’ time–space consumption: A new approach using GPS data and GIS tools. Tour. Geogr..

[32] Edwards D., Dickson T., Griffin A., Hayllar B., Richards G., Munsters W. (2010). Tracking the urban visitor: Methods for examining tourists’ spatial behaviour and visual representations. Cultural Tourism Research Methods.

[33] Zheng W., Zhou R., Zhang Z., Zhong Y., Wang S., Wei Z., Ji H. (2019). Understanding the tourist mobility using GPS: How similar are the tourists?. Tour. Manag..

[34] Park S., Yuan Y., Choe Y. (2021). Application of graph theory to mining the similarity of travel trajectories. Tour. Manag..

[35] D’Urso P., Massari R. (2013). Fuzzy clustering of human activity patterns. Fuzzy Sets Syst..

[36] Nanni M., Pedreschi D. (2006). Time-focused clustering of moving objects. J. Intell. Inf. Syst..

[37] Spaccapietra S., Parent C., Damiani M.L., de Macedo J.A., Porto F., Vangenot C. (2008). A conceptual view on trajectories. Data Knowl. Eng..

[38] Zhu X., Sun T., Yuan H., Hu Z., Miao J. (2019). Exploring group movement pattern through cellular data: A case study of tourists in hainan. Int. J. Geo-Inf..

[39] Ling F., Sun T., Zhu X., Chen Q., Tang X., Ke X. Mining travel behaviors of tourists with mobile phone data: A case study in Hainan. Proceedings of the 2016 2nd IEEE International Conference on Computer and Communications (ICCC).

[40] Kasahara H., Watabe T., Iiyama M. (2021). Tourist transition model among tourist scenic spots based on GPS trajectory. J. Smart Tour..

[41] Pownall I. (2009). Network Analysis and Tourism: From Theory to Practice. Tour. Anal..

[42] Zaheer A., Gozubuyuk R., Milanov H. (2010). It’s the connections: The network perspective in interorganizational research. Acad. Manag. Perspect..

[43] Racherla P., Hu C. (2020). A social network perspective of tourism research collaborations. Ann. Tour. Res..

[44] Wu B., Xiao H., Dong X., Wang M., Xue L. (2012). Tourism knowledge domains: A key word analysis. Asia Pac. J. Tour. Res..

[45] Ye Q., Li T., Law R. (2013). Acoauthorship network analysis of tourism and hospitality research collaboration. J. Hosp. Tour. Res..

[46] Ying T., Xiao H. (2012). Knowledge linkage: A social network analysis of tourism dissertation subjects. J. Hosp. Tour. Res..

[47] Schaffer V., Lawley M. (2012). An analysis of the networks evolving form an artificial reef development. Curr. Issues Tour..

[48] Albrecht J.N. (2013). Networking for sustainable tourismdtowards a research agenda. J. Sustain. Tour..

[49] Larsen T.A., Gujer W. (1997). The concept of sustainable urban water management. Water Sci. Tech..

[50] Balocco C., Grazzini G. (2000). Thermo dynamic parameters for energy sustainability of urban areas. Sol. Energy.

[51] Antrop M. (1998). Landscape change: Plan or chaos?. Landsc. Urban Plan..

[52] Geng H., Gu S., Guo D. (2004). Analysis of the Evolution of Urban Household Energy Consumption Structure Based on Information Entropy. J. Nat. Resour..

[53] Tan Y., Wu C. (2003). Research on the Information Entropy Differentiation Law of Regional Land Use Structure. J. Nat. Resour..

